# Supplementary material for: Low Levels of Low-Density Lipoprotein Cholesterol and Endothelial Function in Subjects without Lipid-Lowering Therapy
Source: J Clin Med. 2020 Nov 24;9(12):3796. doi: 10.3390/jcm9123796 (PMC7761134; doi:10.3390/jcm9123796)
Supplement: Supplementary file 1 [file jcm-09-03796-s001.pdf]

SUPPLEMENTAL MATERIAL

Low Levels of Low-density Lipoprotein Cholesterol and Endothelial Function in Subjects without Lipid-lowering Therapy

Yuji Takaeko, MD;[1] Masato Kajikawa, MD, PhD;[2] Takayuki Yamaji, MD;[1] Takahiro Harada, MD;[1] Yiming Han, MD;[1] Yasuki Kihara, MD, PhD;[1] Eisuke Hida, PhD;[3] Kazuaki Chayama, MD, PhD;[4] Chikara Goto, PhD;[5] Yoshiki Aibara, MS;[6] Farina Mohamad Yusoff, MD;[6] Shinji Kishimoto, MD, PhD;[6] Tatsuya Maruhashi, MD, PhD;[6] Ayumu Nakashima, MD, PhD;[7] Yukihito Higashi, MD, PhD, FAHA[2,6]

[1]Department of Cardiovascular Medicine,
Hiroshima University Faculty of Medicine Graduate School of Biomedical and Health Sciences, Hiroshima, Japan
[2]Division of Regeneration and Medicine, Medical Center for Translational and Clinical Research, Hiroshima University Hospital, Hiroshima, Japan
[3]Department of Biostatistics and Data Science,
Osaka University Faculty of Medicine Graduate School of Medicine, Osaka, Japan
[4]Department of Gastroenterology and Metabolism, Graduate School of Biomedical and Health Sciences, Hiroshima University, Hiroshima, Japan
[5]Department of Physical therapy, Hiroshima International University, Hiroshima, Japan
[6]Department of Cardiovascular Regeneration and Medicine, Hiroshima University Research Institute for Radiation Biology and Medicine, Hiroshima, Japan
[7]Department of Stem Cell Biology and Medicine, Hiroshima University Faculty of Medicine Graduate School of Biomedical and Health Sciences, Hiroshima, Japan

Address for correspondence: Yukihito Higashi, MD, PhD, FAHA
Department of Cardiovascular Regeneration and Medicine,
Research Institute for Radiation Biology and Medicine (RIRBM), Hiroshima University
1-2-3 Kasumi, Minami-ku, Hiroshima 734-8551, Japan
Phone: +81-82-257-5831 Fax: +81-82-257-5831
E-mail: yhigashi@hiroshima-u.ac.jp

Supplemental Table

Table S1. Univariate Analysis of the Relationship between FMD and Variables.

Variables	β	r	p value
Age, yr	-0.343	-0.34	<0.001
Body mass index, kg/m^2	-0.171	-0.17	<0.001
Systolic blood pressure, mmHg	-0.178	-0.18	<0.001
Diastolic blood pressure, mmHg	-0.099	-0.10	<0.001
Heart rate, bpm	-0.024	-0.02	0.039
Total cholesterol, mg/dL	-0.073	-0.07	<0.001
Triglycerides, mg/dL	-0.138	-0.14	<0.001
HDL-C, mg/dL	0.054	0.05	<0.001
LDL-C, mg/dL	-0.069	-0.07	<0.001
Glucose, mg/dL	-0.167	-0.17	<0.001
Framingham risk score, %	-0.245	-0.25	<0.001

FMD indicates flow-mediated vasodilation; HDL-C, high-density lipoprotein cholesterol; LDL-C, low-density lipoprotein cholesterol.

Table S2. Clinical characteristics in propensity score mathced subjects.

Variables	<50 mg/dL (n=35)	≥50 mg/dL (n=35)	p value
Age, yr	48.9±13	49.7±10	0.795
Body mass index, kg/m^2	22.4±4.0	22.4±3.5	0.970
Gender, men/women	28/7	29/6	0.759
Systolic blood pressure, mmHg	126±20	126±18	0.925
Diastolic blood pressure, mmHg	78±13	81±12	0.226
Heart rate, bpm	67±12	68±10	0.583
Total cholesterol, mg/dL	132±23	207±28	<0.001
Triglycerides, mg/dL	87 (65, 168)	116 (77, 172)	0.853
HDL-C, mg/dL	64±21	66±20	0.776
LDL-C, mg/dL	43±6	116±25	<0.001
Glucose, mg/dL	99±12	102±23	0.568
Medications, n (%)			
Anti-hypertensive therapy	10 (28.6)	7 (20.0)	0.403
Anti-hyperglycemic therapy	3 (8.6)	4 (11.4)	0.690
Framingham risk score, %	2.6±2.0	7.8±7.1	<0.001
Medical history, n (%)			
Hypertension	13 (37.1)	13 (37.1)	1.000
Dyslipidemia	12 (34.3)	19 (54.3)	0.092
Diabetes mellitus	3 (8.6)	4 (11.4)	0.690
Smokers	12 (34.3)	11 (31.4)	0.799
FMD, %	6.2±3.5	6.6±2.1	0.570

Data are presented as mena ± SD or median (interquartile range). HDL-C indicates high-density lipoprotein cholesterol; LDL-C, low-density lipoprotein cholesterol; FMD, flow-mediated vasodilation. Variables used for propensity score-matched analysis; age, body mass index, gender, heart rate, glucose, triglycerides, HDL-C, hypertension (yes or no), diabetes mellitus (yes or no), smokers (yes or no), use of anti-hypertensive drugs (yes or no) and use of anti-hyperglycemic therapy (yes or no).

Supplementary Figures

Figure S1. Flow chart of the study design

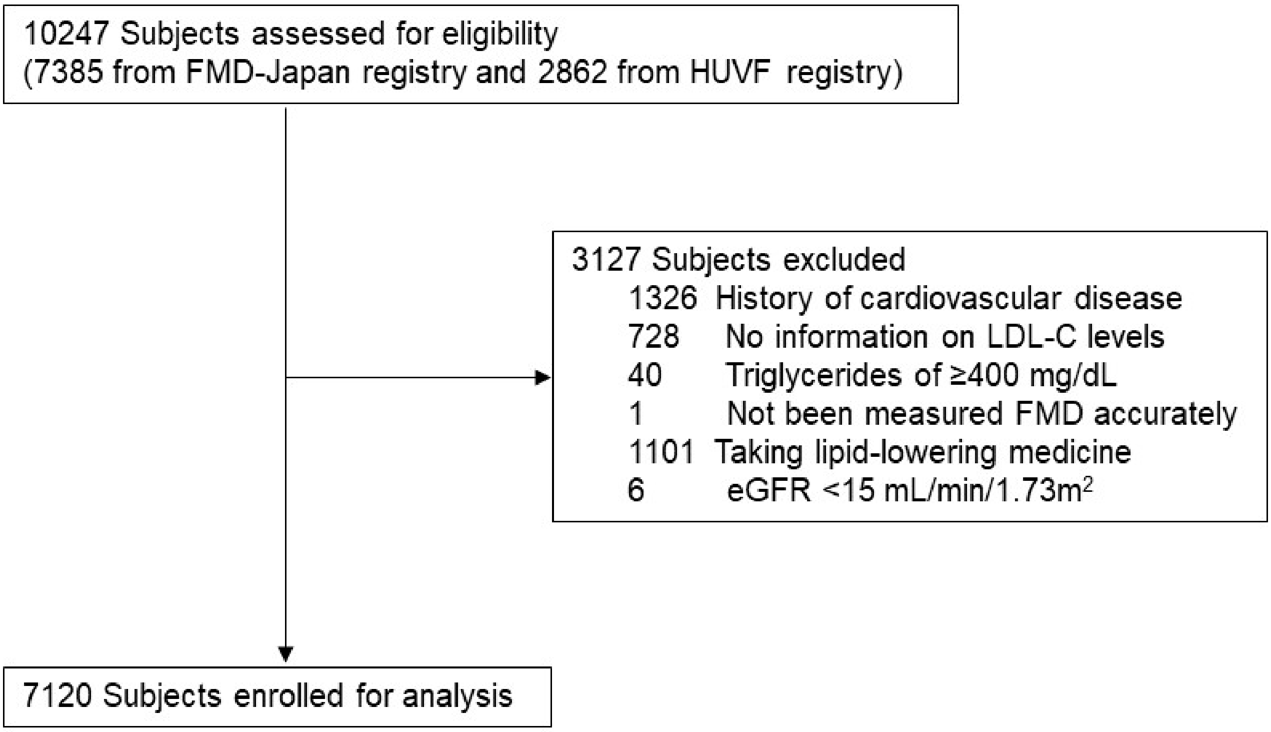

Figure S1. Flow chart of the study design from screening to completion of the study. FMD indicates flow-mediated vasodilation; HUVF, Hiroshima University Vascular Function; LDL-C, low-density lipoprotein cholesterol, eGFR, estimated glomerular filtaration rate.

Figure S2. Comparison of FMD values of each group

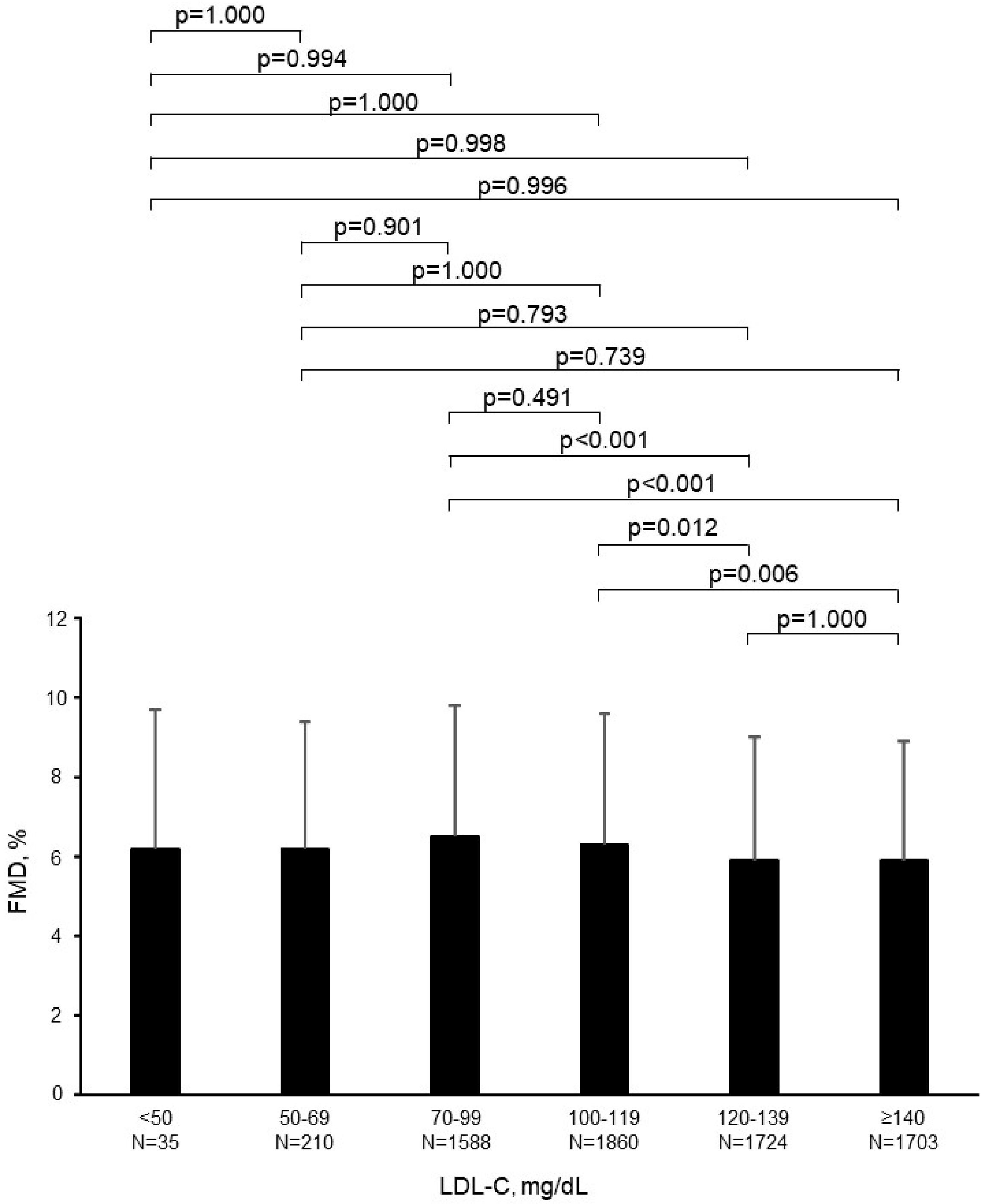

Figure S2. Bar graphs show flow-mediated vasodilation (FMD) in low-density lipoprotein cholesterol (LDL-C) <50 mg/dL, 50-69 mg/dL, 70-99 mg/dL, 100-110 mg/dL, 120-139 mg/dL, and ≥140 mg/dL group. The error bars indicate the standard deviation.
